# Unilateral Blepharoptosis from Renal Cell Carcinoma

**DOI:** 10.15586/jkcvhl.2016.61

**Published:** 2016-12-23

**Authors:** Federico Greco, Lorenzo Sabatino, Francesco Sabatino, Manuele Casale, Carlo Cosimo Quattrocchi, Bruno Beomonte Zobel

**Affiliations:** 1 Unit of Diagnostic Imaging and 2 Unit of Otolaryngology, Università Campus Bio-Medico di Roma, Rome, Italy; 3 Eye Unit, Ashford & St Peter’s Hospitals NHS Foundation Trust, UK

**Keywords:** blepharoptosis, kidney cancer, orbital MRI, ptosis, renal cell carcinoma

## Abstract

Blepharoptosis is the drooping or inferior displacement of the upper eyelid. Blepharoptosis can be either congenital or acquired. Tumour metastasis is one of the acquired causes of blepharoptosis. The lungs, locoregional lymph nodes, bone and liver are the usual sites of metastases of renal cell carcinoma (RCC); however, unusual locations of RCC have also been reported. Herein, we describe a case of a 47-year-old man with unilateral ptosis and blurred vision due to metastatic RCC. We describe the different causes of blepharopstosis, the path that led to the diagnosis, and how RCC can metastasize to unusual anatomical regions such as the orbit. Symptoms such as exophthalmos, lid edema, diplopia, ptosis, cranial nerve paralysis or blurred vision may mime a benign disease; however, they could also be the symptoms of a systemic malignancy.

## Introduction

Blepharoptosis (or simple ptosis) is the drooping or inferior displacement of the upper eyelid (1). It is diagnosed measuring palpebral fissure height, marginal reflex distance, upper eyelid crease and levator function (2). It must also be differentiated by pseudoptosis (3). The causes of ptosis can be classified into two categories: congenital and acquired. Congenital ptosis, which is an anomaly of the muscle elevator development, is unilateral in 69% of cases, but can also be symmetrical or asymmetrical bilateral (4). Other causes of congenital ptosis are the Marcus Gunn jaw-winking syndrome, the Duane’s syndrome and the congenital Horner’s syndrome (5). Acquired ptosis are classified into several subcategories: mechanical, myogenic, neuromuscular, neurogenic and cerebral (5). The most common cause of ptosis is mechanical, specifically aponeurotic (senile or young involution) in which there is bilateral ptosis due to a progressive relaxation or dehiscence of the levator aponeurosis; in young people, the causes are rubbing eyes and prolonged use of contact lenses. Other causes of mechanical ptosis are as follows: inflammation or infiltration of the eyelid (with malignancy or amyloid), dermatochalasis, tumours of the orbit or lid, post-surgical edema, chalazion, a lost contact lens in the superior fornix or a scar (5). Among non-mechanical acquired ptosis, we distinguish the following categories: myogenic (chronic progressive external ophthalmoplegia, myotonic dystrophy and oculopharyngeal muscular dystrophy), neuromuscular (myasthenia gravis and botulism), neurogenic (Horner’s syndrome, oculomotor paresis, Miller–Fisher syndrome and ophthalmoplegic migraine) and cerebral (especially right hemisphere lesions) (5). Another cause of ptosis is tumour metastasis. Herein, we present a case of a 47-year-old man who presented with unilateral ptosis and blurred vision, due to a repetitive lesion that stemmed from metastatic renal cell carcinoma (RCC). Although rare, many studies report RCC metastasis to orbits (6–8).

## Case report

Written, informed consent was obtained from the patient for publication of the report and any accompanying images. A 47-year-old man was admitted to the Unit of Diagnostic Imaging Università Campus Bio-Medico di Roma for a brain magnetic resonance imaging examination. He was referred to our hospital because of narrowing of the left eyelid. The disorder was associated with occasional nuanced blurred vision.

**Figure 1.**
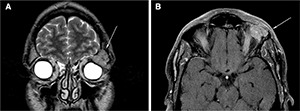
Magnetic resonance imaging: Coronal T2 TSE sequence (**A**) and coronal SE T1 sequence with fat suppression (**B**), showing expansive lesion of probable bone origin with intraorbital development, which appears isointense in T2 and shows contrast enhancement on T1 fat-suppressed sequence (white arrows).

At the level of the left orbit roof in the frontal bone, in-depth thin-film sequences showed a solid lesion likely to be extraconal bone departure, with expansive growth characteristics (diameters of 23 × 15 × 25 mm). This was characterized by intermediate signal intensity on T1- and T2-weighted images, evidence of contrast enhancement and intra-orbital development at the upper-outer quadrant level. The deforming upper-side of the eyeball incorporated the lacrimal gland with no safe cleavage planes from the upper rectus, lateral rectus and the upper eyelid muscle. They were completely incorporated into the lesion (**Figure 1**). Total body computed tomography (CT) showed numerous secondary mediastinal lymphadenopathy that were bilateral hilars (diameters right 13 × 23 mm and left 36 × 23 mm) or subcarinal (diameters 39 × 25 mm).

**Figure 2.**
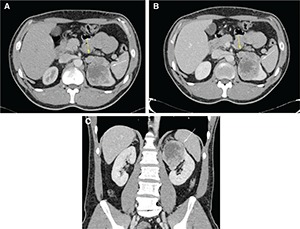
CT: axial scans during arterial (**A**) and venous (**B**) phases and coronal scan during venous phase (**C**) of the abdomen, show expansive lesion, partially exophytic, located in the upper pole of the left kidney (diameters 72 × 56 × 70 mm) with necrosis in the context (white arrows); it also observes repetitive lesion of the right adrenal gland body (diameter 20 mm) (**A** and **B**) (yellow arrows).

At the upper pole of the left kidney was a partially exophytic lesion (diameters 72 × 56 × 70 mm), which marked the upper caliceal group, with contextual necrotic phenomena (**Figure 2**). Locoregional lumbar-aortic lymphadenopathy with a maximum diameter of 20 mm, two repetitive lesions at the body (diameter 21 mm) (**Figure 2A and B**) and medial arm (diameter 10 mm) of the left adrenal gland, and nodulation of 8 mm in diameter in the right adrenal gland were also seen. In addition, multiple bone lesions in skeletal segments were examined. After CT staging, the patient was subjected to endoscopic ultrasound, and multiple specimens at lymph-node stations 7 and 11L were collected for cytology. As a result of cytological sampling, the patient was diagnosed with RCC. The patient was subsequently followed up in another hospital for management.

## Discussion

About 25–33% of RCC patients have metastases at the first diagnosis (9). These are mainly detected in the lungs (75% of cases), regional lymph nodes (65%), bone (40%) and liver (40%). In 15% of cases, metastases to head and neck have been observed. Macroscopically, RCC metastases are highly vascular and soft, elastic and yellowish white in color. Histological features are as follows: encapsulating connective tissue, clear cell borders, copious clear cytoplasm and round or oval nuclei (10). RCC is often noted to invade the local vascularization through the interlobular, arcuate and interlobar veins and metastasize through the systemic circulation (6). Orbital involvement due to metastasis usually presents with orbital mass, exophthalmos, lid edema, ptosis, diplopia and/or cranial nerve paralysis (11). Orbital metastases (OM) are relatively rare with the incidence ranging from 1% to 13% reported series of orbital tumours (12).

A US study, conducted on 100 patients, found a higher percentage of OM from the breast (53%), followed by lung (8%), melanoma (6%) and kidney (5%) (13). An Italian study, performed on 93 patients, found the greatest percentage of mammary origin (36%), followed by kidney (10%), lung (8%) and skin (6%) (14). Another study, carried out in Southern China on 46 patients, showed a higher percentage of nasopharyngeal origin (30%), followed by lung (8%), liver (6%), kidney (4%) and breast (4%) (15). Finally, a Japanese study, performed on 128 patients, found the greatest frequency of pulmonary origin (16%), followed by breast (15%), liver (14%), adrenal neuroblastoma (7%) and kidney (3%) (16).

OM from RCC and clear cell are rare; they can also develop after the removal of the primary tumour (17). RCC promotes angiogenic factors that promote tumour and ocular neovascularization (18). Transitional cell carcinoma of the renal pelvis constitutes about 15–20% of renal tumours in adults and 90% of the renal pelvis tumours; squamous cell cancer and adenocarcinoma constitute the remaining 10% (19). Some cases of metastatic transitional cell tumour have been reported from the bladder to the choroid and orbit, whereas only one case of metastasis to the choroid from transitional cell carcinoma of the renal pelvis is reported (20, 21). Adult Wilms tumour is a rare tumour that tends to appear in advanced stage and has a poor prognosis (22). Only one case of metastasis to the eye (choroidal metastases) from adult Wilms cancer is reported (23).

## Conclusion

Symptoms such as exophthalmos, lid edema, diplopia, ptosis, cranial nerve paralysis or blurred vision could be the symptoms of a systemic malignancy. These must be investigated using available measures. Cytology and histology are invaluable tools in diagnostic pathology (24). In this case, cytology helped to identify metastatic RCC as the cause of unilateral blepharoptosis.
